# Children’s Eating

**Published:** 1890-12

**Authors:** 


### CHILDREN'S EATING.

Some parents compel their children to eat against their will, as when they come to the breakfast table without an appetite, or have lost it in prospect of a visit or a ride, or for the sake of "eating their plates clean" in discouragement of wasteful habits. Unless we are thirsty we cannot drink the purest spring water without aversion, and as for eating when there is no appetite it is revolting, as any one may prove to himself by attempting to take a second meal in twenty minutes after having eaten a regular dinner. The appetite, the hunger, is excited by the presence of gastric juice about the stomach ; but if there is no gastric juice there can be no hunger, no appetite, and to compel a child to swallow food when it is distasteful is an absurdity and a cruelty.

---

THE EGG A REMEDY FOR DYSENTERY.—The egg is considered one of the best remedies for dysentery; beaten up slightly, with or without sugar, and swallowed at a gulp, it tends, by its emolient qualities, to lessen the inflammation of the stomach and intestines, and by forming a transient coating on these organs, to enable nature to resume her health sway over a diseased body. Two, or at most three eggs per day would be all that is required in ordinary cases ; and since eggs are not merely medicine, but food as well, the lighter the diet otherwise and the quieter the patient is kept the more certain and rapid is the recovery

